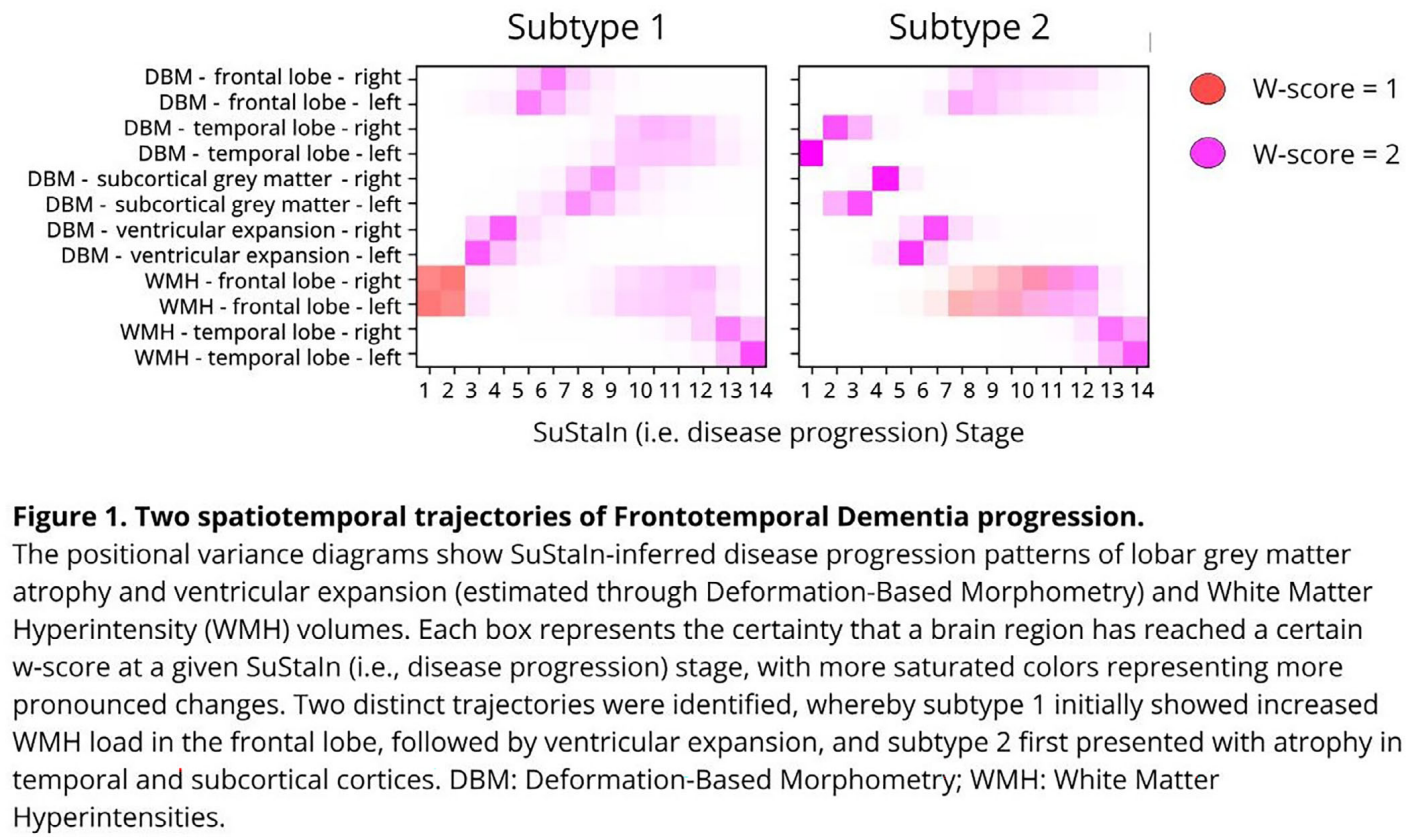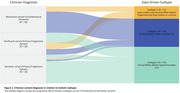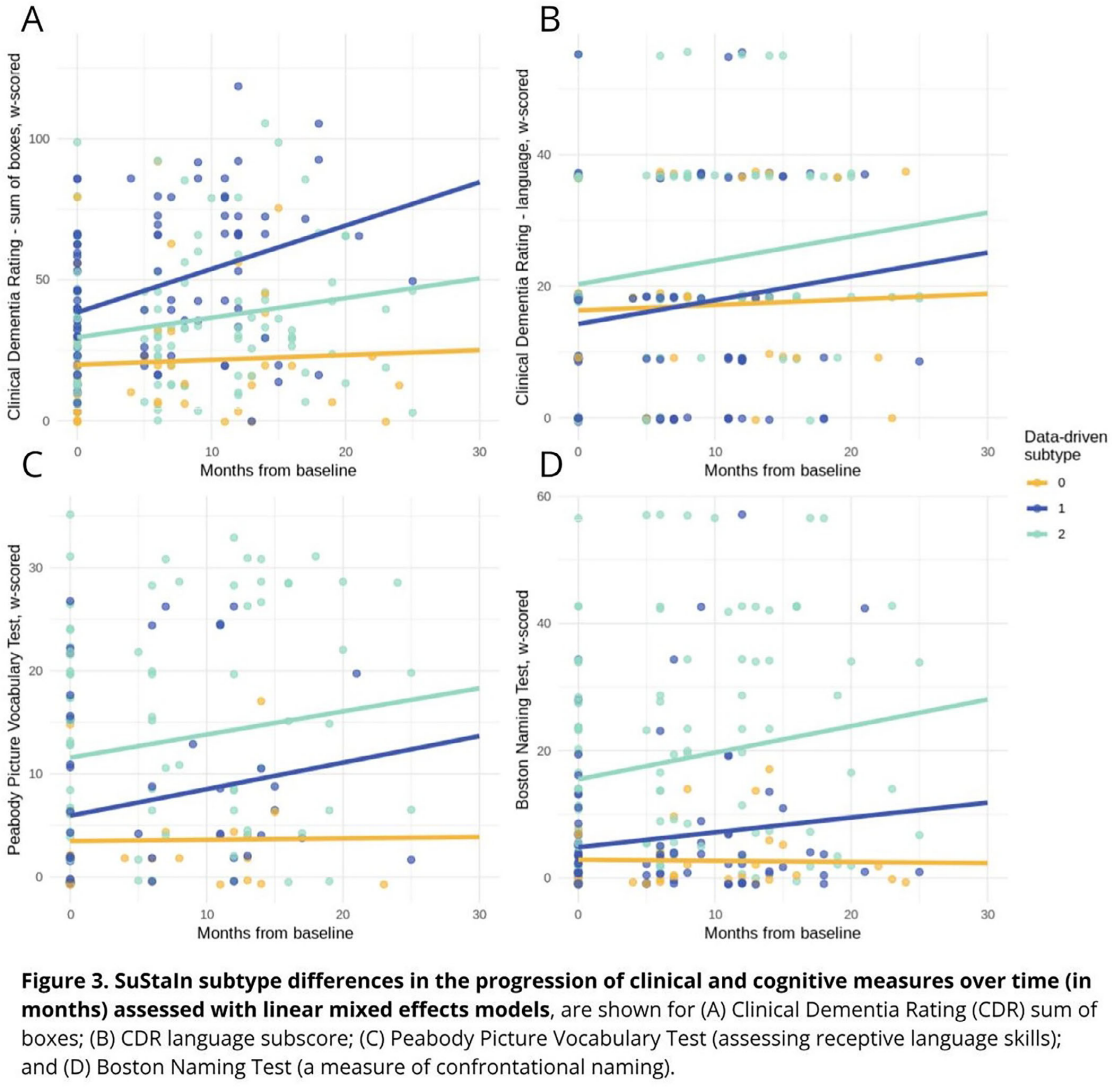# Data‐Driven Characterization of Heterogeneous Brain Atrophy and White Matter Hyperintensity Progression in Frontotemporal Dementia

**DOI:** 10.1002/alz70856_106914

**Published:** 2026-01-08

**Authors:** Amelie Metz, Maxime Montembeault, Yashar Zeighami, Sylvia Villeneuve, Mahsa Dadar

**Affiliations:** ^1^ Douglas Mental Health University Institute, Montréal, QC, Canada; ^2^ Department of Psychiatry, McGill University, Montréal, QC, Canada; ^3^ Douglas Mental Health University Institute, Centre for Studies on the Prevention of Alzheimer's Disease (StoP‐AD), Montréal, QC, Canada

## Abstract

**Background:**

Frontotemporal Dementia (FTD) encompasses a spectrum of neurodegenerative disorders with a diverse range of clinical presentations and overlapping phenotypes, highlighting its heterogeneity. This study applied disease progression modeling to identify novel, data‐driven subtypes of brain atrophy patterns and White Matter Hyperintensity (WMH) burden, as well as their progression, in the FTD spectrum.

**Methods:**

Our analysis included 56 individuals with behavioral variant Frontotemporal Dementia (bvFTD), 33 with semantic variant Primary Progressive Aphasia (svPPA), and 24 with non‐fluent variant Primary Progressive Aphasia (nfvPPA) from the Frontotemporal Lobar Degeneration Neuroimaging Initiative (FTLDNI) cohort. We quantified frontal, temporal, and subcortical brain atrophy and ventricular expansion using Deformation‐Based Morphometry (Metz et al. 2025) and derived frontotemporal lobar WMH volumes based on FLAIR (Dadar et al. 2021). To identify subtypes of participants with distinct brain atrophy and WMH patterns, we employed the Subtype and Stage Inference (SuStaIn) method (Young et al., 2018). Additionally, we examined the differences in trajectories of cognitive decline and brain measures between subtypes using linear mixed‐effects models.

**Results:**

SuStaIn identified three distinct disease progression subtypes within the FTD spectrum. Subtype zero (*n* = 15) showed no increased brain atrophy and WMH burden compared to healthy controls. In the first subtype (*n* = 45), brain atrophy progressed from temporal to subcortical regions, followed by ventricular expansion and eventually increased WMH load in the bilateral frontal lobe. In the second subtype (*n* = 53), frontal lobe WMH involvement preceded ventricular expansion and frontal lobe atrophy (Figure 1).

The atrophy‐first subtype was associated with greater cognitive impairment, particularly in language‐related tasks such as confrontational naming and verbal fluency. In contrast, the WMH‐first subtype exhibited higher behavioral impairment, along with a more rapid decline, as reflected in the Clinical Dementia Rating (all *p* < 0.05, Figure 2). While SuStaIn did not fully distinguish all clinical variants, it effectively differentiated most individuals with svPPA and bvFTD, aligning with the symptom profiles observed in these subtypes (Figure 3).

**Conclusion:**

Our findings suggest distinct disease progression trajectories along the FTD continuum that can be identified in vivo, with subtypes differing in whether WMH burden or brain atrophy precedes the other.